# Power of Topical Blood Supply in Gonorrhoea: Cases I to XXIII

**Published:** 1897-07

**Authors:** Thos. B. Keyes

**Affiliations:** Surgeon and Gynecologist to the Bethesda Free Dispensary, Chicago


### Power of Topical Blood Supply in Gonorrhoea: Cases I to XXIII.

BY THOS. B. KEYES, M. D.,
Surgeon and Gynecologist to the Bethesda Free Dispensary, Chicago.

The primary treatment which I employ is to fill a two-quart fountain syringe with a warm solution of bichloride of mercury, 1 to 40,000, and irrigate the urethra thoroughly, using a No. 9 soft rubber catheter, going slowly and allowing the water to pass by its side so as not to push the virus farther back. The urethra is irrigated about one or two inches farther back than the inflammation goes.

The fountain syringe is again filled, this time with a warm weak solution of sulphate of zinc (1¼ grains to 2 quarts of water), and the urethra again irrigated. After having thoroughly treated the urethra by the irrigations above described, I now fill a Keyes-Ultzman's urethral syringe with bovinine, and inserting this well back of the inflammation, gradually inject, and withdraw the syringe, at the same time. With most cases one such treatment is sufficient. The disease is aborted, and no discharge or soreness remains; the bovinine acting both as a soothing balm, and to feed and build up the lesions of the mucous surface which have taken place.

In no instance has any complication set in after the above treatment, and in those cases where complications had existed, they quickly subsided under the treatment with the usual hygienic precautions.

SUMMARY OF TWENTY-THREE CASES TREATED AS ABOVE.

In sixteen cases: eleven first attacked 1 to 5 days before treatment; and five, second attack 2 to 4 days before treatment; there were no complications, and the cure was accomplished with one treatment.

One case with phimosis, 18 days standing, cured with four treatments.

One with chancroid, second attack 13 days before; cured with three treatments.

Two with balanitis, attacked 7 days before; cured with three treatments.

One with ardor urinæ and chordee, of 9 days standing; cured with three treatments.

Two with lymphangitis, cured with two and three treatments respectively.

CHANCROID ULCERATION: CASE NO. XXIV.

From among particularly bad cases of chancroid—the worst I have ever seen—I report the following: Miss M. W. had treated herself for about two months, when the ulceration began to extend rapidly, and when I saw her the whole right labia majora, and to the extent of about one inch into the vagina, was deeply ulcerated. After putting on a bichloride of mercury pack for about thirty minutes, I gave her some bovinine, which she applied faithfully, and in two weeks the sore was entirely healed.

